# Evaluation of a Viscoelastic Coagulation Monitoring System (VCM Vet^®^) and Its Correlation with Thromboelastometry (ROTEM^®^) in Diseased and Healthy Dogs

**DOI:** 10.3390/ani13030405

**Published:** 2023-01-25

**Authors:** Imke Hennink, Laureen Peters, Geert van Geest, Katja-Nicole Adamik

**Affiliations:** 1 Department of Veterinary Clinical Medicine, Division of Small Animal Emergency and Critical Care, Vetsuisse Faculty, University of Bern, 3012 Bern, Switzerland; 2 Department of Veterinary Clinical Medicine, Clinical Laboratory, Vetsuisse Faculty, University of Bern, 3012 Bern, Switzerland; 3 Interfaculty Bioinformatics Unit, University of Bern, 3012 Bern, Switzerland

**Keywords:** thromboelastometry, VCM, ROTEM, coagulopathy, hemostasis

## Abstract

**Simple Summary:**

Viscoelastic coagulation testing provides an assessment of global coagulation in whole blood, from the beginning of clot formation to clot lysis. A novel bed-side viscoelastic coagulation monitor (VCM) has been developed for use in small animals. The aims of this study were to determine inter-device agreement of two VCM devices, to evaluate the correlation between VCM and rotational thromboelastometry (ROTEM), and to determine the accuracy of VCM to diagnose hypo-, normo-, and hypercoagulability. ROTEM analysis was performed using anticoagulated blood and VCM analysis using native blood. Twenty healthy and forty diseased dogs with and without coagulopathies were enrolled. The VCM inter-device agreement was moderate to strong for most of the parameters. Correlation between VCM and ROTEM was moderate to strong for parameters of clotting time and clot strength. The VCM most likely detects true hypocoagulability and thus can reliably rule out hypocoagulability. The VCM has a high sensitivity for diagnosing normocoagulability, but incorrectly classified dogs with abnormal coagulation as normocoagulable. The VCM was not able to detect hypercoagulability. ROTEM and VCM cannot be used interchangeably.

**Abstract:**

Thromboelastometry provides a real-time assessment of global coagulation in whole blood. A novel bed-side viscoelastic coagulation monitor (VCM) has been developed for use in small animals. The aims of the study were to determine inter-device agreement of two VCM devices, to evaluate the correlation between VCM and rotational thromboelastometry as a reference method (ROTEM), and to determine the sensitivity and specificity of VCM to diagnose hypo-, normo-, and hypercoagulability. ROTEM (extrinsic and intrinsic activation) analysis was performed using citrated blood and VCM analysis using native blood. Twenty healthy and forty diseased dogs with and without coagulopathies were enrolled. The VCM inter-device agreement was moderate to strong for most of the parameters, depending on the grading scale. Correlation between VCM and ROTEM was moderate to strong for parameters of clotting time and clot strength. The VCM most likely detects true hypocoagulability and reliably rules out hypocoagulability. The VCM has a high sensitivity in diagnosing normocoagulability, but incorrectly classified dogs with abnormal coagulation as normocoagulable. The VCM was not able to detect hypercoagulability. ROTEM and VCM cannot be used interchangeably.

## 1. Introduction

A viscoelastic blood analysis provides a dynamic in vitro assessment of global coagulation, from the beginning of clot formation to fibrinolysis in whole blood [1]. The analysis includes the interaction of all components of coagulation, including platelets, red blood cells, fibrin, coagulation factors, and thrombin. The two most often used viscoelastic technologies are thromboelastography (TEG) and rotational thromboelastometry (ROTEM) [1,2]. Both TEG and ROTEM assess the kinetics of clot formation and dissolution and clot strength by measuring and displaying the amount of a continuously applied rotational force that is transmitted to an electromechanical transduction system by the developing clot [3]. In the ROTEM system, a cylindrical cup containing a whole blood sample remains fixed in a heating block while a pin suspended on a ball-bearing mechanism oscillates. The subsequent rotation of the pin is inversely related to the viscoelastic clot strength and is visualized in the typical TEG curve [3]. ROTEM is commonly used with citrated blood, which is re-calcified to activate clotting at the time the test is initiated (Appendix A, Table A1). By using an automated pipette, the blood, and different activators (e.g., tissue factor (ExTEM), ellagic acid (InTEM)) are delivered to the cup [2,3]. Non-activated tests are available (NaTEM) but are not commonly used. The standard ROTEM device can analyze 4 samples simultaneously. The clinical use of ROTEM is still limited by factors such as costs, efforts (no bed-side test), and the required technical knowledge and skills. For that reason, viscoelastic tests are predominantly found at academic centers. Recently, a novel portable bed-side viscoelastic coagulation monitor (VCM) was developed, which utilizes glass surface activation of untreated whole blood [4]. Three hundred μL of native blood is delivered to a test cartridge, which is then placed in the VCM machine. Due to the large contact area between the drawn blood and the glass inside the cartridge (contact activation), no other activators are necessary. As in ROTEM, a curve is generated by the machine and the duration of the test also lasts 60 min (Appendix A, Table A1). A good-to-moderate agreement in test results between ROTEM NaTEM (non-activated method) and the VCM in people undergoing major surgery was found [5]. 

Recently, reference intervals for VCM in cats [6], dogs [7], and mice [8] have been established. In addition, in healthy cats, no significant correlation between VCM and TEG was found [6]. In dogs, direct correlations of VCM values with TEG parameters were not performed due to the narrow range of the normal values and the need to evaluate patients with a wide range of hemostatic abnormalities [7]. Moreover, the study of Wang et al. found an acceptable rate of agreement between two VCM devices used in healthy dogs during the peri-anesthetic period [9].

To the authors’ best knowledge, no veterinary studies currently exist that compare VCM and ROTEM in dogs. Therefore, the aims of our study were to determine inter-device agreement of two VCM devices in a heterogenous groups of dogs, to evaluate the correlation between VCM and ROTEM (ExTEM and InTEM), and to determine the sensitivity and specificity of the VCM to diagnose hypo-, normo-, and hypercoagulability.

## 2. Materials and Methods

This prospective study was performed at the Small Animal Clinic, Department of Clinical Veterinary Medicine, Vetsuisse Faculty, University of Bern, Switzerland, between November 2019 and July 2021, and included 20 healthy and 40 diseased dogs. According to “Measurement Procedure Comparison and Bias Estimation Using Patient Samples” (Third Edition), a minimum of 40 samples is required to evaluate the bias between two methods that measure the same analyte. Ideally, 50% of the samples should be outside the reference intervals and therefore a total animal number of 60 dogs (20 healthy and 40 diseased) was chosen and duplicate measurements of each sample were performed [10]. The study was approved by the Animal Experiment Committee of the Swiss Federal Veterinary Office (registration number BRE95/19), and informed owner consent was obtained for all dogs prior to inclusion.

### 2.1. Animals 

#### 2.1.1. Healthy Dogs

Staff-owned dogs and blood donors ≥1 year of age and ≥5 kg of body weight served as a healthy control group. Dogs were eligible if they had no history or evidence of recent or chronic medical conditions and had not received any medication, except for routine preventative healthcare, within the preceding 6 months [11,12,13]. Dogs were classified as healthy based an unremarkable physical examination, and unremarkable complete blood count (CBC), serum chemistry and conventional coagulation parameters.

#### 2.1.2. Diseased Dogs

The diseased group consisted of client-owned dogs with suspected and/or confirmed hyper- or hypocoagulability, which were presented to the emergency service, or which were already hospitalized. For the definition of coagulation status, the reader is referred to Section 2.5.

### 2.2. Blood Sampling

Blood sampling was performed by atraumatic jugular venipuncture using a 21G needle attached to a 10 mL syringe and a total of 6 mL blood was taken [14]. In dogs with suspected hypocoagulability, blood was taken from the cephalic or saphenous vein. A volume of 350 μL whole blood each was immediately delivered to the VCM cartridge for immediate analysis (see below). The remaining blood was transferred into one EDTA tube (1.3-mL), one Li-heparin tube (1.3 mL), and two 3.8% (106 mM) sodium citrate tubes (1.3 mL each). The EDTA and the Li-heparin tubes were used to perform complete blood cell counts (Advia 2120i, Siemens Healthcare Diagnostics AG, Zurich, Switzerland) and plasma biochemistry panels (Cobas c501, Roche Diagnostics, Rotkreuz, Switzerland), respectively. One citrated tube was used to perform a conventional coagulation test (Start MAX, Stago CH SA, Zurich, Switzerland), which consisted of prothrombin time (PT), activated partial thromboplastin time (aPTT), and fibrinogen concentration analyses (Clauss method). All analyses were performed according to the standards of the Clinical Diagnostic Laboratory, Department of Clinical Veterinary Medicine, Vetsuisse Faculty, University of Bern. The remaining citrated tube was used to perform two ROTEM tests in parallel.

### 2.3. ROTEM Analysis

The ROTEM analysis (ROTEM^®^, TEM Innovations GmbH, Munich, Germany) was performed within 20 min after blood sampling. A blood volume of 300 μL of citrated whole blood is needed for each channel. Each sample was extrinsically (ExTEM, Tem International GmbH, Munich, Germany) and intrinsically (InTEM, Tem International GmbH, Munich, Germany) activated (Appendix A, Table A1), according to the manufacturer’s instructions. Duplicate measurements were performed in parallel on the same machine. Data collected included CT, CFT, alpha, MCF, A10, A20, LY30 and LY45 (Appendix A, Table A1).

### 2.4. VCM Analysis

For VCM analysis (VCM Vet^®^, Entegrion, Durham, North Carolina 27703) a volume of 350 μL whole blood each was immediately transferred into the pre-heated (37 °C) VCM cartridges and analysis was immediately started according to the manufacturer’s instructions. Data collected included CT, CFT, alpha, MCF, A10, A20, Li30 and Li45 (Appendix A, Table A1). The VCM analysis was performed in duplicate on two devices simultaneously. System check cartridges were used as per the manufacturer’s instructions at least once weekly and quality control kits using premade lyophilized human plasma samples were run once monthly to ensure proper functioning of the analyzer.

### 2.5. Definition of Coagulation Status 

To define hypo-, normo-, and hypercoagulability, institutional reference intervals for ROTEM and VCM were generated (in 20 healthy dogs). For ROTEM, the mean of each duplicate measurement of the respective parameter in InTEM or ExTEM was used. For VCM, the mean of the duplicate measurements from the two instruments was used. 

ROTEM and VCM coagulation status was defined based on CT, CFT and MCF as follows [14,15]:

1. Normocoagulable: all parameters of interest within the reference range.
2. Hypocoagulable: ≥2 hypocoagulable parameters (CT/CFT prolonged, or MCF decreased) in ExTEM or InTEM.
3. Hypercoagulable: ≥2 hypercoagulable parameters (CT/ CFT shortened, or MCF increased) in ExTEM or InTEM.

### 2.6. Statistical Analysis 

All statistical analyses were performed with R version 4.0.4 [16]. All parameters were assessed for normality using the Kolmogorov–Smirnov test. For the establishment of institutional reference intervals for the VCM and ROTEM in healthy dogs, the parametric method with the function refLimit of the R package reference intervals [17], according to the ASVCP reference interval guidelines [18], was used. To determine the reproducibility of the measurements (inter-device reproducibility for VCM and inter-channel reproducibility for ROTEM) Lin’s concordance correlation coefficient (Lin’s CCC) was analyzed. The following scale of strength of the correlation was used: 0.00–0.10 negligible, 0.10–0.39 weak, 0.40–0.69 moderate, 0.70–0.89 strong, 0.90–1.00 very strong [19]. For determination of the correlation between ROTEM (ExTEM and InTEM) and VCM test results, Spearman correlations were calculated with the R base function cor.test. P-values for differences between two dependent correlation coefficients were calculated with the R package cocor [20], using the statistical test for comparing dependent correlations of Hittner et al. (2003) [21]. Statistical differences between hypocoagulable and normocoagulable patients by means of standard coagulation groups were calculated with a student’s t-test assuming an unequal variance between groups. Online MedCalc software [22] was used to calculate the sensitivity and specificity.

## 3. Results

### 3.1. Animals

Demographic data are summarized in Table 1. The diseases within the diseased group are listed in Appendix B, Table A4.

### 3.2. Measured Values and Institutional Reference Intervals 

Median and interquartile range of all measured values in normo-, hypo- and hypercoagulable dogs are summarized in Appendix A, Table A2. Institutional reference intervals (RIs) for ROTEM and VCM are presented in Table 2.

### 3.3. Inter-Device and Inter-Channel Agreement

In ROTEM, the strength of agreement between the two channels was very strong for CT (ExTEM only), CFT, 𝛼-angle, MCF, A10 and A20 (Table 3). In VCM, the strength of inter-device agreement was very strong for CT, 𝛼-angle, MCF, A10, and A20 (Table 3).

### 3.4. Correlation between Equivalent ROTEM and VCM Parameters

Spearman correlations for equivalent ROTEM and VCM parameters are presented in Table 4. There was a strong correlation between ROTEM and VCM test results for CT (InTEM), A10 and A20 (ExTEM, InTEM), and MCF (ExTEM, InTEM); a moderate correlation between the two systems for CFT (ExTEM, InTEM) and 𝛼-angle (InTEM); and a weak correlation between the two systems for CT, 𝛂-angle and LI45 (ExTEM). 

### 3.5. Coagulations States and Sensitivity and Specificity of VCM for Diagnosing Different Coagulation States against ROTEM 

Forty-four (InTEM) and forty-seven (ExTEM) dogs, respectively, were diagnosed to be normocoagulable in ROTEM (Appendix A, Table A2). Of these, 39 dogs were concordantly normocoagulable in VCM, resulting in an agreement of coagulation status in 83% (ExTEM) and 89% (InTEM) of normocoagulable dogs, respectively. Likewise, twelve (InTEM) and ten (ExTEM) dogs, respectively, were diagnosed to be hypocoagulable in ROTEM (Appendix A, Table A2). Of these, eight dogs were concordantly hypocoagulable in VCM, resulting in an agreement of coagulation status in 67% (InTEM) and 80% (ExTEM) of hypocoagulable dogs. Only 3 dogs were hypercoagulable in ROTEM and one dog in VCM, but there was no agreement between the ROTEM and VCM in detecting hypercoagulability. The coagulation states and agreements between the ROTEM and VCM, with the corresponding sensitivity and specificity, are presented in Table 5. Measured values of laboratory parameters in dogs that were concordantly normo- or hypocoagulable in all three tests (ROTEM and VCM) are presented in Appendix A, Table A3. 

## 4. Discussion

This study aimed to determine inter-device agreement of two VCM devices in dogs, to evaluate the correlation of equivalent parameters between VCM and ROTEM, and to determine the sensitivity and specificity of VCM to diagnose normo-, hypo- and hypercoagulability in dogs with different coagulations states. Both devices perform a real-time viscoelastic measurement by using whole blood and creating a curve during clot formation. As a first step in our study, inter-device correlation was evaluated, and institutional reference intervals were established in a small group of dogs. Except for CFT and LI, a very strong to strong inter-device correlation for VCM for all other parameters was found in our study, with Lin’s CCC between 0.90 to 0.93. This corroborates the results from Buriko et al. [7], who found strong to moderate Lin’s correlations for all parameters. Since identical parameters were measured in the same blood sample, a much stricter grading could have been applied, with r > 0.99 as perfect agreement, 0.99 to 0.95 as substantial, 0.95 to 0.90 as moderate, and <0.90 as poor agreement [23]. With this grading, the VCM reaches not more than moderate strength of agreement between the two devices in our study. In agreement with Buriko et al., we recommend establishing unique reference intervals for the respective device. The weak inter-device correlation regarding LI30 and LI45 in our study is due to the fact that LI 30 and LI 45 have a small range (the values are mostly exactly 100%), but sometimes a high measurement error between the two samples occur (there were single samples with very strong deviation, such as 60% in one device vs. 100% in the other device). Regarding ROTEM, inter-channel correlations were generally better than for VCM. 

Indeed, due to significant differences between device systems in terms of blood samples (native vs. citrated), sample processing (syringe vs. automated pipette), clot activation (no activators vs. various activators), and technology (cup and pin vs. glass plates) [4], no direct agreement between equivalent ROTEM and VCM parameters is expected in the present study. Nevertheless, in our study, a strong correlation (Spearman correlation r > 0.70) was found between ROTEM and VCM for CT (InTEM), MCF (InTEM, ExTEM), A10 and A20 (InTEM, ExTEM) and a weak correlation for CFT and 𝛼-angle (InTEM). Similarly, Brearton et al. found a strong correlation (Spearman correlation r > 70) between ROTEM NATEM (non-activated ROTEM) and VCM for CT, A10, A20, and MCF in subjects undergoing major surgery [5]. Finding a better correlation between InTEM and VCM for some parameters could possibly be explained by the fact that both systems test the intrinsic pathway. Nonetheless, it is generally accepted that the results of different viscoelastic coagulation tests (e.g., ROTEM and TEG) are not interchangeable; this is also true for ROTEM and VCM [24] and both, although similar technologies, should be considered individual systems. 

The goal of a bed-side whole blood coagulation test is the determination of coagulation status to guide clinical judgement and therapy. For this purpose, diseased dogs were categorized as normo-, hypo- or hypercoagulable using ROTEM as a reference, and the sensitivity and specificity of VCM against ROTEM in diagnosing different coagulation states was analyzed. An excellent agreement in coagulation status was found between VCM and ExTEM in hypocoagulable dogs with a sensitivity and specificity of 90% and 94%, respectively. This means that VCM most likely detects true hypocoagulability and thus reliably rules out hypocoagulability. Furthermore, VCM is very sensitive in diagnosing normocoagulability (94% for ExTEM-normocoagulability and 91% for InTEM normocoagulability), but specificity is moderate to poor, falsely classifying dogs with abnormal coagulability as normocoagulable in 23–44% of cases when compared to ExTEM and InTEM, respectively. Therefore, hypo- and hypercoagulable states may be missed, and a normocoagulable VCM result should ideally be followed up by more established methods. Furthermore, VCM was unable to diagnose hypercoagulability. The diagnostic performance regarding hypercoagulability could not be conclusively evaluated in our study, as in total only 3 dogs were classified as hypercoagulable.

Diagnosis of hypo- or hypercoagulability in our study was based on two or more parameters outside the institutional reference range in ROTEM. However, there is inconclusive evidence on how hypo- or hypercoagulability should be defined in companion animals based on TEG or ROTEM parameters [14,15] and it is also controversial to consider ROTEM as the gold standard. Besides the PROVETS guidelines, there are veterinary studies that created combinations of the suggested PROVETS guidelines to classify hyper- or hypocoagulability, using ROTEM variables alone [25] or combined with plasmatic coagulation parameters [26]. In addition, it is described to use the G-value (actual measure of clot strength), for determining hypo- or hypercoagulable states [14,27]. The G-value can be calculated manually (G measured in Kdynes/cm^2^ = [(5000 × MCF)/(100 − MCF)]) or is calculated by the respective device. 

In our study, we did not only compare two different viscoelastometric devices, but also citrated vs. native whole blood. Citrated whole blood has been shown to differ from native fresh whole blood with regards to coagulant properties. Studies found a hypercoagulable viscoelastic hemostatic test response with citrated blood compared to native fresh whole blood [28,29,30]. To overcome this pre-analytical difference, ROTEM NaTEM (TRUE-Non-Activated ROTEM) analysis could have been performed, where no coagulation activators or reagents are added to the blood sample for analysis and analyses needs to be started within 4 min [31]. However, this was not performed in our study due to feasibility reasons. There are advantages and disadvantages for the use of native fresh whole blood. Native blood samples will reflect the true coagulation profile of the patient. It avoids some of the artifacts that citrate can induce in a blood sample, such as the antiaggregatory effect on platelets and underestimation of heparin, and does not need recalcification [28]. Further, even slight over- or under-filling of citrated test tubes will induce a pre-analytic error in the respective testing, which is not the case in native blood samples. On the other hand, according to the VCM manufacturer’s instructions, the native whole blood sample must be applied into the cartridge within 4 min. Therefore, the use of native whole blood limits the time to analyze the sample to minutes and the device must be located close to the patient. The use of the same type of blood (either native or citrated for both systems) probably lead to comparisons that are more reliable between different viscoelastometric devices. Recently, citrated whole blood samples were used with the VCM device in 10 dogs and citrated sample results remained consistent up to 4 h after blood collection [32]. 

Our study has several limitations. First, only 12 dogs fell into the hypocoagulable category. Thus, only 12 dogs were available to analyze the performance of the VCM for the detection of hypocoagulability. This low number is not very representative. Contrary to our expectations, a large proportion of diseased dogs did not fall into the abnormal coagulation category and further acquisition of more patients would have been necessary. However, due to insufficient time and funds we were not able to acquire more dogs. Second, no reliable statement can be made about the detection of hypercoagulability because only 3 dogs fell into this category. Third, as mentioned above, native and anticoagulated samples were compared. Ideally, samples should be the same to rule out preanalytical differences in the samples.

## 5. Conclusions

The VCM inter-device agreement was moderate to strong for most of the parameters. Correlation between VCM and ROTEM was moderate to strong for parameters of clotting time and clot strength. VCM most likely detects true hypocoagulability and reliably rules out hypocoagulability. Furthermore, VCM has a high sensitivity in diagnosing normocoagulability, but incorrectly classified dogs with abnormal coagulation as normocoagulable compared with ExTEM or InTEM, respectively. Further studies on the performance of VCM in populations with specific coagulopathies are required.

## Figures and Tables

**Table 1:** Patient demographics.

	Diseased (n = 40)	Healthy (n = 20)
**Breed Group (n)**		
Herding dogs and cattle dogs	3	6
Pinscher and Schnauzer—Molossoid and Swiss Mountain and Cattledogs	9	2
Terrier	3	3
Spitz and primitive types	1	0
Scent hounds and related breeds	2	0
Pointing Dogs	5	1
Retrievers—Flushing Dogs—Water Dogs	5	3
Companion and Toy Dogs	5	0
Crossbreeds	7	5
**Sex**		
Female castrated [n (%*)]	10 (16.7)	7 (11.7)
Female intact [n (%*)]	8 (13.3)	5 (8.3)
Male castrated [n (%*)]	12 (20.0)	5 (8.3)
Male intact [n (%*)]	10 (16.7)	3 (5.0)
Age [median (range)] years	7.6 (0.6 to 14.1)	6.3 (1.0 to 10.8)

Breed groups according to Fédération Cynologique Internationale available on http://www.fci.be/en/ (accessed on 27 December 2022). *, percent of the whole population.

**Table 2:** Institutional reference intervals for ROTEM and VCM based on results of 20 healthy dogs.

Device						
**ROTEM**	**CT (s)**	**CFT (s)**	**Alpha (°)**	**MCF (mm)**	**A10 (mm)**	**A20 (mm)**
ExTEM	30−53	60−155	63−80	55−77	41−68	50−73
InTEM	125−218	52−148	65−81	55−73	40−62	49−69
**VCM**	**CT (s)**	**CFT (s)**	**Alpha (°)**	**MCF (vcm units)**	**A10 (vcm units)**	**A20 (vcm units)**
	194−492	107−230	49−69	26−46	18−32	23−40

CT, clotting time; CFT, clot formation time; alpha, alpha angle; MCF, maximum clot firmness; A10, clot strength after 10 min; A20, clot strength after 20 min; s, seconds; °, degree; mm, millimeters; vcm units, specific units used by the VCM.

**Table 3:** Inter-device (VCM) and inter-channel (ROTEM InTEM and ExTEM) agreement in blood samples of 60 dogs (20 healthy dogs and 40 diseased dogs).

	VCM			ROTEM InTEM			ROTEM ExTEM		
Parameter	Lin’s CCC	Lower CI	Upper CI	Lin’s CCC	Lower CI	Upper CI	Lin’s CCC	Lower CI	Upper CI
**CT**	0.91	0.85	0.94	0.86	0.77	0.91	0.97	0.95	0.98
**CFT**	0.80	0.70	0.86	0.97	0.95	0.98	0.99	0.99	1.00
**𝛂-angle**	0.93	0.89	0.96	0.95	0.91	0.97	0.98	0.96	0.99
**MCF**	0.91	0.85	0.95	0.97	0.95	0.98	0.90	0.83	0.94
**A10**	0.93	0.89	0.96	0.97	0.96	0.99	0.99	0.98	0.99
**A20**	0.93	0.88	0.96	0.99	0.98	0.99	0.99	0.98	0.99
**LI30**	0.19	−0.07	0.42	0.88	0.87	0.89	*NA*	*NA*	*NA*
**LI45**	0.36	0.11	0.56	0.60	0.49	0.69	0.16	−0.01	0.33

Scale for the strength of concordance correlation coefficient agreement between scores: 0.00–0.10 negligible, 0.10–0.39 weak, 0.40–0.69 moderate, 0.70–0.89 strong, 0.90–1.00 very strong [17]. NA, not applicable; CT, clotting time; CFT, clot formation time; α-angle, alpha angle; MCF, maximum clot firmness; A10, clot strength after 10 min; A20, clot strength after 20 min; LI30, clot lysis after 30 min; LI45, clot lysis after 45 min.; s, seconds; °, degree; mm, millimeters; vcm units, specific units used by the VCM; Lin’s CCC, Lin’s concordance correlation coefficient; CI, confidence interval.

**Table 4:** Spearman correlations between equivalent ROTEM and VCM parameters of 60 dogs.

VCM	ROTEM	Spearman	*p*-Value
CT	InTEM-CT	0.71	<0.0001
	ExTEM-CT	0.11	0.419
CFT	InTEM-CFT	0.61	<0.0001
	ExTEM CFT	0.57	<0.0001
𝛼-angle	InTEM-𝛼-angle	0.52	<0.0001
	ExTEM-𝛼-angle	0.28	0.032
MCF	InTEM-MCF	0.79	<0.0001
	ExTEM-MCF	0.80	<0.0001
A10	InTEM-A10	0.79	<0.0001
	ExTEM-A10	0.83	<0.0001
A20	InTEM-A20	0.82	<0.0001
	ExTEM-A20	0.85	<0.0001
LI30	InTEM-LY30	−0.13	0.321
	ExTEM-LY30	0.05	0.716
LI45	InTEM-LY45	0.03	0.844
	ExTEM-LY45	0.18	0.185

CT, clotting time; CFT, clot formation time; α-angle, alpha angle; MCF, maximum clot firmness; A10, clot strength after 10 min; A20, clot strength after 20 min; LI30, clot lysis after 30 min.; LI45, clot lysis after 45 min; s, seconds; °, degree; mm, millimeters; vcm units, specific units used by the VCM.

**Table 5:** Cross table presenting the agreement of different coagulation states between ROTEM and VCM (ROTEM as the reference device).

	VCM Norm	VCM Hypo	VCM Hyper
ExTEM norm (n = 47)	n = 44	n = 2	n = 1
ExTEM hypo (n = 10)	n = 1	n = 9	n = 0
ExTEM hyper (n = 3)	n = 2	n = 1	n = 0
Sensitivity	93.6%	90.0%	n/a
Specificity	76.9%	94.0%	n/a
InTEM norm (n = 44)	n = 40	n = 3	n = 1
InTEM hypo (n = 12)	n = 4	n = 8	n = 0
InTEM hyper (n = 3)	n = 3	n = 0	n = 0
Sensitivity	90.9%	66.7%	n/a
Specificity	56.3%	91.7%	n/a

Norm, normocoagulable; hypo, hypocoagulable; hyper, hypercoagulable; n, number of tested dogs.

## Data Availability

Data are available on request.

## Appendix A

**Table A1:** Measurement techniques and parameters for coagulation analyses with ROTEM and VCM.

	ROTEM	VCM
**Technology**	A cylindrical cup containing whole blood remains fixed while a pin suspended on a ball-bearing mechanism oscillates every 6 sec through application of a constant force. As the clot strength increases the rotation of the pin is impeded and is detected optically using a charge coupled device image sensor system.	Test cartridge consists of two parallel glass plates, with a small gap. The blood sample lies between the two plates and they slide past each other with controlled velocity, creating a shear stress between the plates. Driving signals of various amplitudes and frequencies can elicit motion between the two plates. An optical system then detects the resulting motion on the sample.
**Sample**	Usually citrated blood (300 μL)	Native blood (350 μL)
**Blood sample processing**	Automated pipette	Directly from syringe
**Activators ** **(only the ones used in this study)**	ExTEM^®^: activation by tissue factor. Assessment of factors VII, X, V, II, I, platelets, fibrinolysis InTEM^®^: activation via the contact phase (phosphor-lipid and ellagic acid). Assessment of factors XII, XI, IX, VIII, X, V, II, I, platelets, fibrinolysis	None (contact activation by high surface area of glass plates)
**Parameters**	CT (s): clotting time (time from start of the measurement until onset of clotting). CFT (s): clot formation time (time between onset of clotting and a clot of 20 mm amplitude). α (°): alpha-angle (angle between midline and tangent to the curve drawn from the 1 mm wide point; describes clot formation kinetics). MCF (mm): maximum clot firmness (maximum amplitude of the curve). A10/A20 (mm): amplitude after 10 and 20 min, respectively (=clot firmness after 10 and 20 min). LY30/LY45 (%): clot lysis at 30 and 45 min, respectively (the amplitude of the clot at 30 and 45 min after clot time; percentage of MCF).	CT (s): clot time (time from beginning of the test until the time when an amplitude of 1% above the baseline is achieved). CFT (s): clot formation time (time between 1% amplitude and 10% amplitude of the clotting signal). α (°): alpha-angle (angle between the time axis and the tangent to the clotting curve through 1% amplitude point; describes clot formation kinetics). MCF (VCM units): maximum clot formation (maximum amplitude reached before clot lysis occurs; firmness of the clot). A10/A20 (VCM units): amplitude at 10 and 20 min, respectively (=clot firmness after 10 and 20 min after clot time). LI30/LI45 (%): lysis index at 30 and 45 min, respectively (amplitude of the clot at 30 and 45 min after clot time; percentage of MCF).

**Table A2:** Median (interquartile range) of ROTEM (ExTEM and InTEM), VCM and laboratory parameters in all dogs classified in normo-, hypo-, and hypercoagulable.

Parameter						
ExTEM	norm	n	hypo	n	hyper	n
CT (RI: 30−53 s)	40 (36–50)	47	467 (401–775)	10	34 (34–36)	3
CFT (RI: 60−155 s)	86 (62–111)	46	141 (75–160)	10	23 (21–30)	3
Alpha (RI: 63−80°)	75 (69–80)	46	43 (28–56)	10	86 (85–86)	3
MCF (RI: 55−77 mm)	68 (63–74)	46	36 (32–37)	10	82 (81–85)	3
A10 (RI: 41−68 mm)	58 (49–65)	45	23 (17–25)	10	81 (78–83)	3
A20 (RI: 50−73 mm)	65 (58–71)	45	31 (25–31)	10	82 (80–85)	3
HKT (RI: 0.28–0.47 L/L)	0.45 (0.36–0.50)	47	0.42 (0.38–0.44)	10	0.37 (0.33–0.41)	3
PLT (RI: 180–520 × 10^9^/L)	224 (141–312)	47	51 (34.50–119.50)	10	663 (452–-668)	3
PT (RI: 5.7–8.5 s)	8.5 (7.95–9.3)	43	12.4 (9.8–17.3)	8	13.0 (12.9–15.9)	3
aPTT (RI: 9.6–14.3 s)	13.3 (12.6–16.5)	43	22.3 (20.0–26.9)	9	7.9 (7.55–8.05)	3
F-gen (RI: 109–311 mg/dL)	301 (188–450)	43	122 (58–232)	7	523 (485–894)	3
**InTEM**	**norm**	**n**	**hypo**	**n**	**hyper**	
CT (RI: 125−218 s)	207 (172−310)	47	447 (324−612)	12	132 (128−151)	3
CFT (RI: 52−148 s)	85 (65−107)	41	455 (367−673)	12	43 (43−50)	3
Alpha (RI: 65−81°)	73 (70−77)	41	38.5 (27−52)	12	82 (81−82)	3
MCF (RI: 55−73 mm)	66 (61−69)	41	36.5 (32−45)	12	74 (72−80)	3
A10 (RI: 40−62 mm)	55 (48−60)	41	22 (19−25)	12	63 (63−73)	3
A20 (RI: 49−69 mm)	63 (56−67)	41	31 (25−35)	12	70 (69−78)	3
HKT (RI: 0.28–0.47 L/L)	0.46 (0.38−0.50)	42	0.39 (0.28−0.43)	12	0.36 (0.33−0.41)	3
PLT (RI: 180–520 × 10^9^/L)	236(166−314)	42	49 (37−140))	12	152 (148−412)	3
PT (RI: 5.7–8.5 s)	8.4 (7.9−9.1)	42	11.0 (9.65−12.8)	10	7.9 ^§^	1
aPTT (RI: 9.6–14.3 s)	13.2 (12.6−16.1)	42	22.2 (16.9−31.1)	11	13 ^§^	1
F-gen (RI: 109–311 mg/dL)	300 (182−447)	41	226 (76−360)	10	1264 ^§^	1
**VCM**	**norm**	**n**	**hypo**	**n**	**hyper**	**n**
CT (RI: 194−492 s)	427 (344−534)	47	609 (479–849)	12	368 *	1
CFT (RI: 107−230 s)	161 (136.3−198)	46	716 (521–1332)	12	91 *	1
Alpha (RI: 49−69°)	55 (48−61)	46	27 (17–32)	12	67.8 *	1
MCF (RI: 26−46 vcm units)	36 (33−41)	46	18 (15–20)	12	51.2 *	1
A10 (RI: 18−32 vcm units)	26 (23−28)	46	9 (7–11)	12	38.4 *	1
A20 (RI: 23−40 vcm units)	32 (30−35)	46	14 (10–16)	12	46.4 *	1
HKT (RI: 0.28–0.47 L/L)	0.45 (0.36−0.50)	47	0.39 (0.37–0.42)	12	0.35 *	1
PLT (RI: 180–520 × 10^9^/L)	230 (148−314)	47	62 (40-122)	12	313 *	1
PT (RI: 5.7–8.5 s)	8.5 (8.0−9.3)	47	12.4 (7.9–18)	10	7.1 *	1
aPTT (RI: 9.6–14.3 s)	13.2 (12.6−15.9)	43	22.3 (19.5–33.2)	11	13.1 *	1
F-gen (RI: 109–311 mg/dL)	301 (188−456)	43	162 (60–443)	9	358 *	1

Norm, normocoagulable; hypo, hypocoagulable; hyper, hypercoagulable; n, number of dogs; CT, clotting time; CFT, clot formation time; Alpha, alpha angle; MCF, maximum clot firmness; A10, clot strength after 10 min; A20, clot strength after 20 min; s, seconds; °, degree; mm, millimeters; units, specific units used by the VCM; HKT, hematocrit; PLT, platelet concentration; PT, prothrombin time; aPTT, activated thromboplastin time; f-gen, fibrinogen; §, value from single measurement of one dog; *, mean from duplicate measurement in one dog; RI, reference interval.

**Table A3:** Median (interquartile range) of laboratory parameters of dogs that were concordantly normo- or hypocoagulable in all three tests (ROTEM and VCM).

Parameter				
ExTEM	norm	n	hypo	n
CT (RI: 30−53 s)	40 (36−50)	39	144 (106−168)	8
CFT (RI: 60−155 s)	88 (67−111)	38	615 (422−777)	8
Alpha (RI: 63−80°)	74 (69−78)	38	40 (26−52)	8
MCF (RI: 55−77 mm)	68 (63−74)	38	34 (32−36)	8
A10 (RI: 41−68 mm)	57 (50−65)	37	21 (17−24)	8
A20 (RI: 50−73 mm)	64 (59−71)	37	28 (5−31)	8
HKT (RI: 0.28–0.47 L/L)	0.46 (0.41−0.50)	39	0.40 (0.36−0.43)	8
PLT (RI: 180–520 × 10^9^/L)	252 (186−321)	39	46 (27−108)	8
PT (RI: 5.7–8.5 s)	8.4 (8.0−8.9)	37	12.4 (9.1−17.0)	6
aPTT (RI: 9.6–14.3 s)	13.2 (12.6−15.0)	37	22.3 (21.1−41.6)	7
F-gen (RI: 109–311 mg/dL)	284 (182−434)	37	91 (57−265)	6
**InTEM**	**norm**	**n**	**hypo**	**n**
CT (RI: 125−218 s)	192 (167−291)	39	406 (285−552)	8
CFT (RI: 52−148 s)	85 (68−107)	37	535 (440−905)	8
Alpha (RI: 65−81°)	73 (70−77)	37	35 (27−50)	8
MCF (RI: 55−73 mm)	66 (61−68)	37	34 (30−36)	8
A10 (RI: 40−62 mm)	53 (48−59)	37	21 (17−23)	8
A20 (RI: 49−69 mm)	61 (56−66)	37	27 (23−30)	8
HKT (RI: 0.28–0.47 L/L)	0.46 (0.41–0.50)	39	0.40 (0.36−0.43)	8
PLT (RI: 180–520 × 10^9^/L)	252 (186−321)	39	46 (27−108)	8
PT (RI: 5.7–8.5 s)	8.4 (8.0−8.9)	37	12.4 (9.1−17.0)	6
aPTT (RI: 9.6–14.3 s)	13.2 (12.6−15.0)	37	22.3 (21.1−41.6)	7
F-gen (RI: 109–311 mg/dL)	284 (182−434)	37	91 (57−265)	6
**VCM**	**norm**	**n**	**hypo**	**n**
CT (RI: 194−492 s)	403 (317−513)	39	508 (452−740)	8
CFT (RI: 107−230 s)	151 (136−183)	38	787 (592−1332)	8
Alpha (RI: 49−69°)	58 (50−62)	38	25 (16−29)	8
MCF (RI: 26−46 vcm units)	36 (33−41)	38	18 (15−19)	8
A10 (RI: 18−32 vcm units)	26 (23−28)	38	9 (7−10)	8
A20 (RI: 23−40 vcm units)	32 (30−35)	38	13 (10−14)	8
HKT (RI: 0.28–0.47 L/L)	0.46 (0.41–0.50)	39	0.40 (0.36−0.43)	8
PLT (RI: 180–520 × 10^9^/L)	252 (186−321)	39	46 (27−108)	8
PT (RI: 5.7–8.5 s)	8.4 (8.0−8.9)	37	12.4 (9.1−17.0)	6
aPTT (RI: 9.6–14.3 s)	13.2 (12.6−15.0)	37	22.3 (21.1−41.6)	7
F-gen (RI: 109–311 mg/dL)	284 (182−434)	37	91 (57−265)	6

Norm, normocoagulable; hypo, hypocoagulable; n, number of dogs; CT, clotting time; CFT, clot formation time; Alpha, alpha angle; MCF, maximum clot firmness; A10, clot strength after 10 min; A20, clot strength after 20 min; s, seconds; °, degree; mm, millimeters; units, specific units used by the VCM; HKT, hematocrit; PLT, platelet concentration; PT, prothrombin time; aPTT, activated thromboplastin time; f-gen, fibrinogen; RI, reference interval.

## Appendix B

**Table A4:** List of diseases within the diseased group.

Disease
**Vector-borne diseases ** Ehrlichiosis **(n = 1);** Leishmaniosis **(n = 2) ** **Gastrointestinal/hepatic/pancreatic disease ** **AHDS (n = 1);** pancreatitis **(n = 1); liver failure (n = 2) ** **Renal disease ** GN **(n = 3); AKI (n = 3) ** **Hemoabdomen ** trauma **(n = 1); s** pontaneous **(n = 1)** **Respiratory disease ** pulmonary fibrosis **(n = 2);** aspiration pneumonia **(n = 2);** pneumonia **(n = 2) ** **Hematologic disease ** Evan’s syndrome **(n = 1);** primary **IMHA (n = 1); ITP (n = 1);** thromboembolic disease **(n = 4)** **Endocrinologic disease ** hyperadrenocorticism **(n = 1) ** **Others ** snake envenomation **(n = 1);** sepsis **(n = 8)**

n, number of dogs; AHDS, acute hemorrhagic diarrhea syndrome, AKI, acute kidney injury; GN, glomerulonephritis; IMHA, immune mediated hemolytic anemia; ITP, immune mediated thrombocytopenia.
